# Clinical use of Gafchromic EBT4 film for in vivo dosimetry for total body irradiation

**DOI:** 10.1002/acm2.14574

**Published:** 2024-11-29

**Authors:** Emily Draeger, Fada Guan, Min‐Young Lee, Dae Yup Han, William Donahue, Zhe (Jay) Chen

**Affiliations:** ^1^ Department of Therapeutic Radiology Yale University School of Medicine New Haven Connecticut USA; ^2^ Department of Radiation Physics MD Anderson Cancer Center Houston Texas USA; ^3^ Department of Medical Physics Memorial Sloan Kettering Cancer Center New York New York USA

**Keywords:** EBT4, gafchromic film, in vivo dosimetry, TBI, total body irradiation

## Abstract

**Purpose:**

In vivo dosimetry is a common requirement to validate dose accuracy/uniformity in total body irradiation (TBI). Several detectors can be used for in vivo dosimetry, including thermoluminescent dosimeters (TLDs), diodes, ion chambers, optically stimulated luminescent dosimeters (OSLDs), and film. TLDs are well established for use in vivo but required expertise and clinical system availability may make them impractical for multifractionated TBI. OSLDs offer quick readout, but recalls have restricted their use. The purpose of this work was to validate the newly available Gafchromic EBT4 film for TBI in vivo dosimetry.

**Methods:**

Film calibration curves were created under standard conditions (6MV/15MV, 1.5/3.0 cm depth, 100 cm source‐to‐surface distance (SSD), 10 × 10 cm^2^ field), and films were scanned at several time points (0.5–24 h) to determine the shortest development time that yielded accurate dose measurements. 4 × 4 cm^2^ films were placed under 1.5 cm thick bolus on the anterior and posterior sides of a solid water phantom to measure entrance and exit dose under TBI conditions (∼600 cm SSD, 39.5 × 39.5 cm^2^ field, 6 MV/15 MV). These measurements were compared to ion chamber and diode readings for validation. Film measurements were also compared to OSLD measurements for three TBI patients.

**Results:**

The shortest development time that resulted in accurate dosimetry and allowed for adequate physician review time was 4 h (± 4% dose accuracy). Film entrance and exit dose measurements were within ± 3.8% of ion chamber and diode readings for 6MV and 15MV beams. Patient film measurements were within ∼ ± 5% for the majority of anatomical measurement locations; however, film and OSLD readings for some anatomic locations deviated by > 10%.

**Conclusions:**

These results indicate that EBT4 film can be utilized for accurate in vivo dosimetry for TBI patients and shows good agreement with diode and ion chamber measurements. Further investigation into film and OSLD differences was not possible due to OSLD recalls.

## 1 INTRODUCTION

Total body irradiation (TBI) is used as part of the conditioning regimen for hematopoietic stem cell transplantation for the treatment of diseases such as multiple myeloma, lymphoma, and leukemia.^1^ The goal of TBI is to deliver a uniform (within ±10%) dose to the whole body, with treatment generally prescribed to midplane at umbilicus. Many TBI treatments are still carried out using traditional extended source‐to‐surface distance (SSD) setups and patient‐specific compensators designed to homogenize the dose over the patient.^2^, ^3^, ^4^, ^5^, ^6^, ^7^ Due to the use of these custom treatment devices, in vivo dosimetry on the first treatment fraction of a multifractionated TBI is imperative to ensure the delivered dose accuracy and uniformity matches the treatment plan.

Several detectors have been utilized for in vivo dosimetry of TBI patients, including ion chambers,^8^ semiconductor diodes,^9^ thermoluminescent dosimeters (TLDs),^10^ Gafchromic film,^11^ and optically stimulated luminescent dosimeters (OSLDs).^12^ Each of these systems is capable of dosimetric accuracy and reproducibility within 5%.^13^ However, there are pros and cons to the use of each of these systems for in vivo dosimetry.

Ion chambers can be used for real‐time in vivo dosimetry; however, these chambers require high voltage for operation and placing them on or around patients may pose electrical risks. Semiconductor diodes can also be used for real‐time in vivo dosimetry. However, depending on the TBI technique and diode system in use, diode cables can be cumbersome while positioning and re‐positioning the patient. Diodes can also suffer from temperature effects when in prolonged contact with the patient,^9^ and require frequent validation due to changes in response caused by accumulated dose. TLDs are small and come in many different forms,^14^ making them ideal for in vivo dosimetry. However, accurate dosimetry requires expertise in proper calibration and handling, and TLD systems may not be widely available for all clinical users. This can limit their use for techniques such as multifractionated TBI.

OSLDs, similar to TLDs, are very small, making them ideal for in vivo dosimetry. OSLDs can be read out after at least 10 min post irradiation,^14^ making them ideal for time‐sensitive clinical applications such as TBI. Several authors have reported accurate in vivo dosimetry results for TBI utilizing OSLDs.^12^, ^15^, ^16^ However, with manufacturer recalls, OSLDs are no longer available for patient measurements. Film is another alternative to OSLDs. Film is nearly tissue equivalent and provides high spatial resolution, it can be used for absolute dose measurements with proper calibration,^17^ and it can be cut to different sizes for different applications. The main downside of film is that current recommendations generally advocate for waiting 24 h after irradiation to scan film for analysis.^17^ Su et al. published on a technique of utilizing film for in vivo dosimetry, and scanning films 6 h after irradiation, which yielded dose measurements within ±5%.^11^

Our clinic has been impacted by the OSLD recall, and while we do have a back‐up diode system for in vivo dosimetry, the purpose of this study was to characterize the use of the newly available Gafchromic EBT4 film (Ashland, Bridgewater, NJ) for use in TBI in vivo dosimetry, by comparing film measurements with our commissioned OSLD (prior to recall) and diode systems with phantom and patient measurements.

## 2 MATERIALS AND METHODS

For this study, Landauer NanoDot OSLDs (Landauer, Glenwood, IL), Sun Nuclear RF‐IVD2 diodes (Sun Nuclear, Melbourne, FL), and Gafchromic EBT4 film (Ashland, Bridgewater, NJ) measurements were compared to ion chamber measurements under TBI irradiation conditions. A PTW TN30013 ion chamber (PTW, Freiburg, Germany) and a PTW Unidos Romeo electrometer (both with valid ADCL calibration) were used for validation of other dosimetry systems. The linear accelerator used for TBI treatment was a Varian TrueBeam (Varian Medical Systems, Palo Alto, CA). Output calibration was completed using the American Association of Physicists in Medicine's (AAPM) TG‐51 protocol.^18^

### 2.1 TBI treatment technique

**FIGURE 1:**
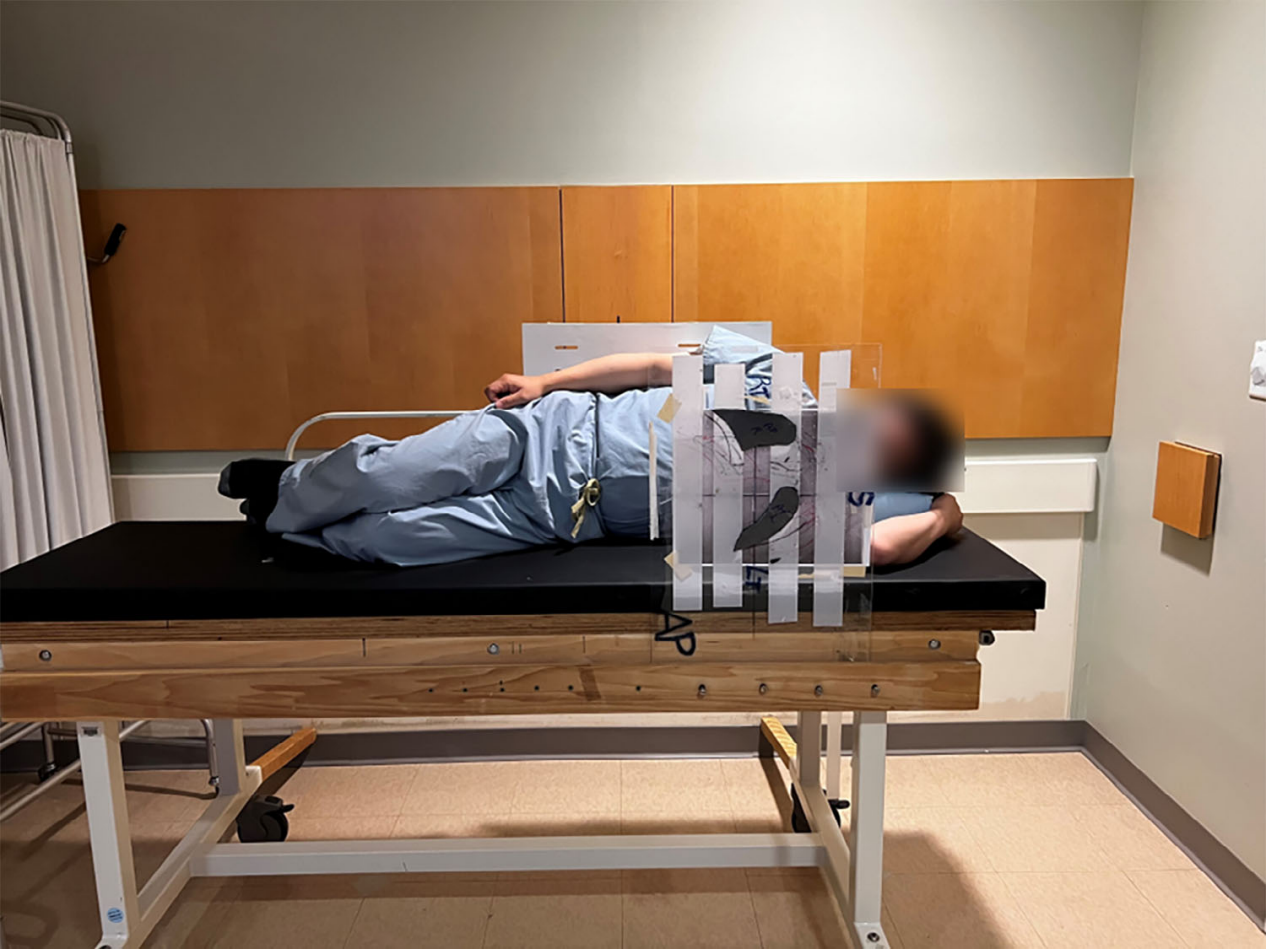
Illustration of patient setup in the left decubitus position for TBI treatment.

At our facility, TBI is carried out using an extended SSD technique, where the patient is placed approximately 600 cm from the source, against a far wall of the linear accelerator vault. The patient is positioned in the left decubitus position, facing the gantry, and the gantry is rotated to approximately 87° to align the beam central axis with the patient's umbilicus. A collimator rotation of 315° is used to provide the largest field size possible to ensure adequate patient coverage. The TBI treatment couch is held off the wall by a fixed distance using 45.7 cm wooden spacers. These spacers are used for all patient treatments. The couch has a wooden backboard that allows the patient to be strapped into a reproducible position, with their back flat against the board, knees bent, left arm bent and resting under their head, and right arm at their side, as in Figure 1. The patient remains in this position for the delivery of the anterior‐posterior (AP) field, and is then turned to a right decubitus position, with the couch remaining in place, for delivery of the posterior‐anterior (PA) field. In the right decubitus position, the patient's stomach is against the wooden backboard, their right arm is bent and resting under their head, and their left arm is at their side. Partial transmission lung blocks composed of lead are used for multi‐fraction treatments to reduce the risk of radiation pneumonitis, and all patients are treated with a custom copper compensator placed at the head of the machine, which is individualized to each patient and is used to modulate the beam intensity over the length of the patient. In vivo dosimetry is carried out at our institution on the first fraction of multi‐fraction TBI treatments by placing dosimeters at six anatomic locations along the patient's body: forehead, neck, umbilicus, right thigh, right knee, and right ankle.

TBI treatments are carried out using energies of 6 MV and 15 MV. A 1 cm thick polymethyl methacrylate spoiler, positioned approximately 10 cm from the patient surface, is used for patients receiving treatment with 15 MV to increase surface dose. No spoiler is used for the majority of patients receiving treatment with 6 MV. Fractionation schemes used in the department depend on patient disease and the conditioning regimen chosen for treatment, but include 200 cGy in one fraction, 600 cGy in three fractions, and 1200 cGy in eight fractions. Treatments delivered in multiple fractions are delivered twice per day, with at least 6 h between the two fractions.

The TBI technique was calibrated using a charge‐to‐dose conversion under standard conditions which was then related to charge measurements under TBI conditions. Using the TG‐51 protocol, the machine output was set to 1.000 cGy/MU at d_max_ (1.5 cm for the 6 MV beam and 3.0 cm for the 15 MV beam) for a 10 × 10 cm^2^ field at 100 cm SSD with gantry and collimator both set to 0°. With a cross‐calibrated PTW2 ion chamber (PTW, Freiburg, Germany) inserted into a solid water phantom (Sun Nuclear, Melbourne, FL, formerly Gammex), 200 MU was delivered using a 10 × 10 cm^2^ field size at 100 cm SSD. For this irradiation setup, total solid water phantom thickness was 22.5 cm. The ion chamber reading was used to determine the charge‐to‐dose conversion under standard conditions. This same solid water setup was then used to collect the charge from 400 MU under TBI conditions, with a 600 cm SSD, 39.5 × 39.5 cm^2^ field size, gantry rotation of 87.5° and collimator rotation of 315°. This yielded a charge per MU value for the TBI geometry. The charge‐to‐dose conversion was then used to convert the charge per MU value to a dose per MU value under TBI conditions. Equation 1 shows the charge‐to‐dose conversion,

(1) $$\frac{D_{\mathrm{TBI}}}{\mathrm{MU}}=D_{0}\times \frac{{\mathrm{MU}}_{\mathrm{STD}}}{q_{\mathrm{STD}}}\times \frac{q_{\mathrm{TBI}}}{{\mathrm{MU}}_{\mathrm{TBI}}}$$

where D_0_ is the calibrated machine output (1.000 cGy/MU at d_max_), MU_STD_ is the MU delivered under standard irradiation conditions (100 cm SSD and 10 × 10 cm^2^ field size), q_STD_ is the charge collected for the delivery of MU_STD_, q_TBI_ is the charge collected under TBI conditions, and MU_TBI_ is the MU delivered under TBI conditions. This was used to determine the entrance dose rate for TBI (at depths of 1.5 cm for 6 MV and 3.0 cm for 15 MV), with a similar method used to determine the exit dose rate for TBI (at depths of 21.0 cm for 6 MV and 19.5 cm for 15 MV).

### 2.2 Diode in vivo dosimetry

**FIGURE 2:**
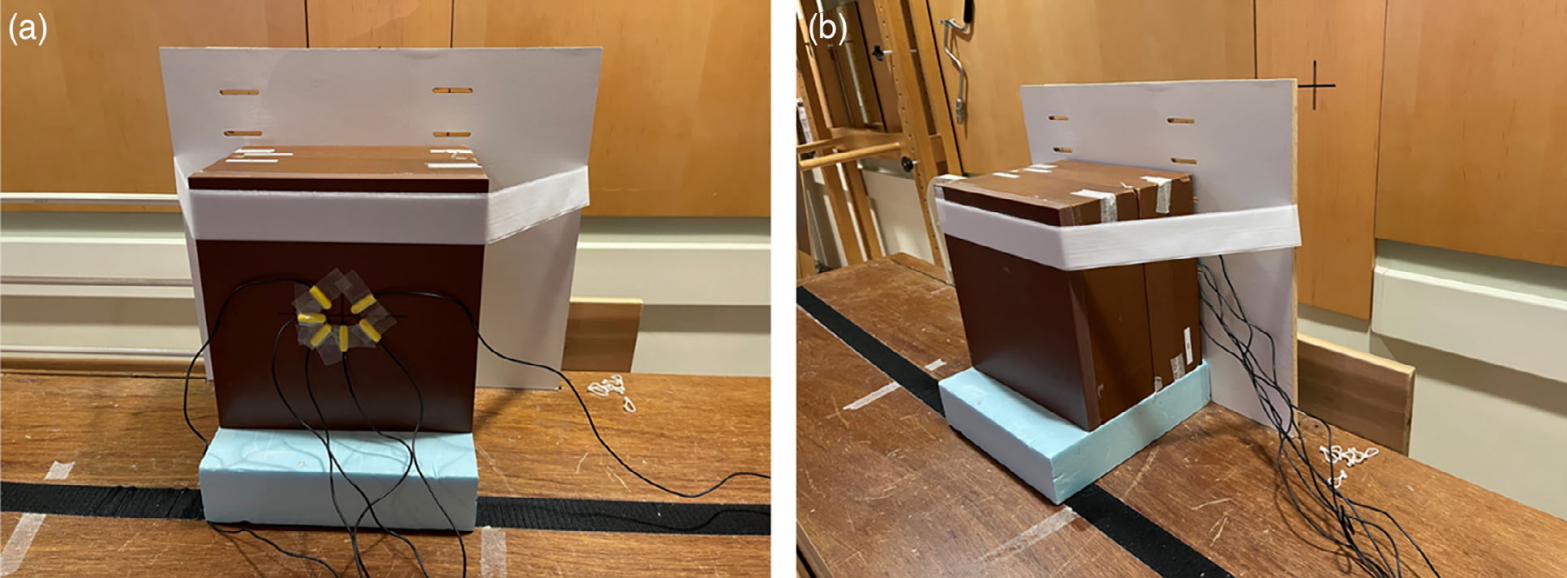
Illustration of setup used for diode measurements under TBI irradiation conditions for entrance dose measurement (2a) and exit dose measurement (2b).

Once the TBI entrance and exit dose rates were determined, the Sun Nuclear RF‐IVD2 diode system (Sun Nuclear, Melbourne, FL) was calibrated for entrance and exit dose measurements under TBI conditions for both clinically used beam energies. Prior to diode calibration, machine output was verified using solid water and an ion chamber following our clinic's monthly QA procedure. Diode calibration was completed by adhering the six measurement diodes to a solid water phantom (22.5 cm thick) using the TBI geometry as described in Section 2.1. This setup is illustrated in Figure 2a for entrance dose measurements and Figure 2b for exit dose measurements. The diodes were adhered to the front of the phantom for entrance measurements, and the back of the phantom for exit measurements. Using the entrance and exit dose rates determined from the charge‐to‐dose conversion presented in Section 2.1, the expected dose was determined for a fixed delivery of 800 MU. Diode readings were acquired at the TBI dose rate (300 MU/min) and related to the expected dose. Prior to each patient measurement, the diode system is set up using this same procedure, and the diode calibration is verified. If the diode readings are found to be outside of 5% of the expected dose, the system is re‐calibrated prior to patient treatment. The system was commissioned and is calibrated using the TBI dose rate to avoid dose‐rate response uncertainties, and no temperature correction is used for diode measurements, as internal validation and literature reviews have indicated temperature dependence for this system is fairly minimal.^9^

### 2.3 OSLD validation

**FIGURE 3:**
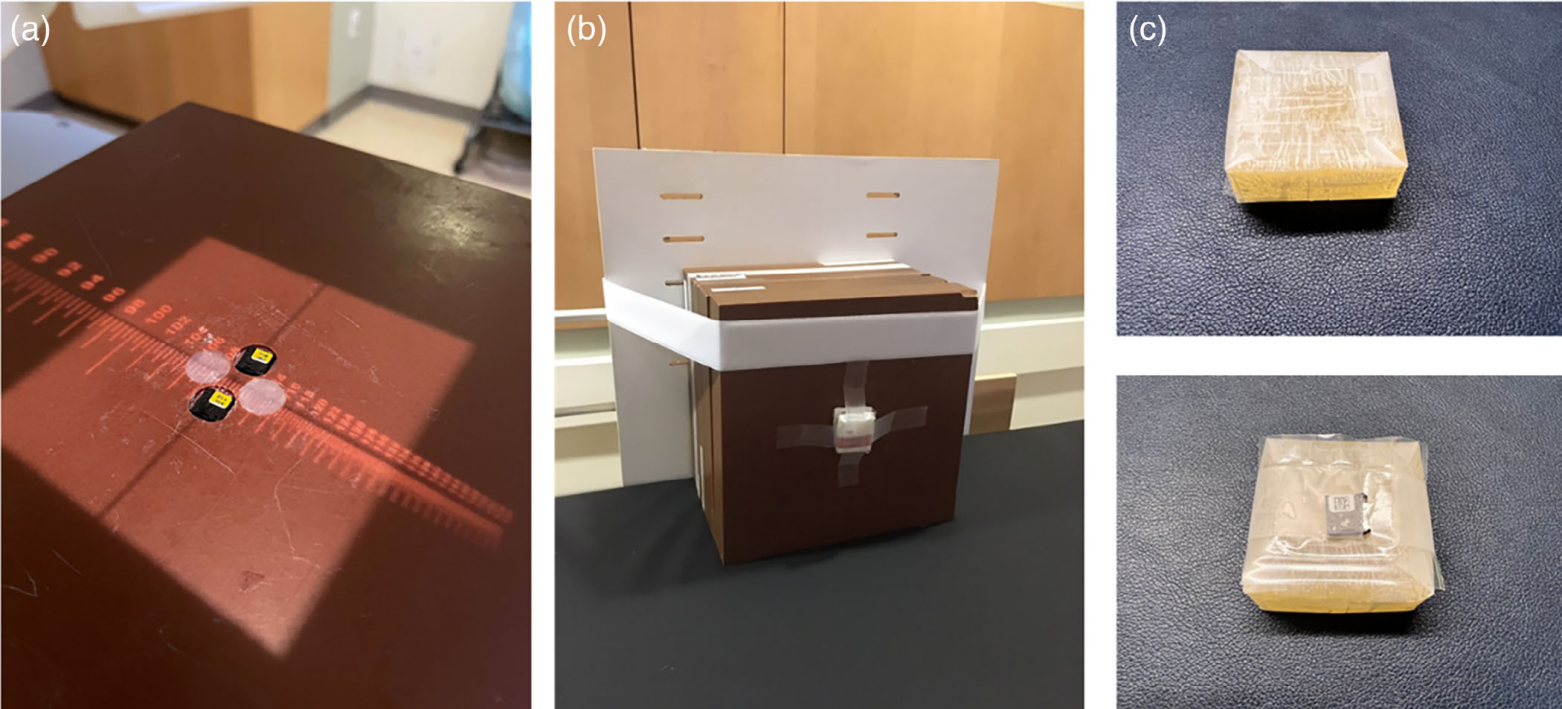
Figure 3a illustrates the setup used to irradiate OSLDs for the creation of the calibration curve. Image illustrates custom phantom with OSLD inserts prior to addition of final buildup and setting of irradiation SSD. 3b illustrates setup for irradiation under TBI entrance dose conditions. Figure 3c shows a top and bottom view of a custom buildup block used for TBI in vivo dosimetry, with an OSLD attached to the bottom of the block.

Extensive testing was completed prior to clinically releasing the OSLD system for use in TBI in vivo dosimetry. All measurements were completed using screened OSLDs, and machine output was verified using our clinic's monthly QA procedure prior to calibration and validation. First, a calibration curve was created using a solid water phantom (10 cm backscatter, 1 cm OSLD insert, 1.5 cm buildup), as shown in Figure 3a. The calibration curve was created for 6 MV, using standard setup conditions (10 × 10 cm^2^ field, 100 cm SSD). Calibration dose values were chosen to span the range of doses expected from TBI and other in vivo dosimetry applications. Calibration doses ranged from 0 to 300 cGy, and two disparate dose points (20 and 150 cGy) were chosen for validation of the calibration curve. Separate OSLDs were also irradiated with the 15 MV beam to validate the use of the 6 MV calibration curve with this higher beam energy.

Following calibration, measurements were taken in the TBI geometry using a solid water phantom (the same phantom setup as described in Section 2.1) to verify the accuracy of entrance and exit dose measurements with OSLDs (Figure 3b). Machine output, thus entrance and exit dose, was verified for both beam energies prior to OSLD irradiation. OSLDs were embedded into the solid water phantom at d_max_, and 1.5 cm or 3 cm from the posterior surface of the phantom, depending on beam energy. These measurements were compared to the expected doses based on our TBI entrance and exit dose calibrations. These OSLD readings were further validated by comparison to diode measurements obtained in the same geometry.

Finally, custom buildup blocks (4 cm × 4 cm × 1.5 cm) were created from 1.5 cm thick bolus to provide buildup for the OSLDs for in vivo measurements (Figure 3c). OSLD readings obtained with these blocks were compared to OSLD readings obtained in solid water to validate the use of these blocks for patient measurements.

AAPM's TG‐191 report^14^ provides details for OSLD angular dependence, temperature dependence, and dose rate dependence. Since these dependencies were found to be minimal (with angular dependence minimal for *en face* irradiation), they were not measured as part of this validation process.

Routine quality assurance testing was also implemented after clinical release of the OSLD system to ensure accuracy of each OSLD batch and the OSLD reader. This included monthly constancy dosimeter readout, irradiation of a subset of detectors from each new batch to validate the reader calibration curve, and annual calibration curve validation using a subset of clinical OSLDs.

### 2.4 Film validation

Calibration curves were created for the EBT4 film by cutting the film into 2.5 cm wide strips, and irradiating each strip to a different dose, from 0 to 1000 cGy, following the standard film calibration protocol established in our clinic.^19^ Film strips were placed on 10 cm of solid water to provide backscatter and covered with either 1.5 cm or 3 cm of solid water for buildup (depending on beam energy). The solid water phantom was set up to 100 cm SSD, and irradiation was completed with a 10×10 cm^2^ field and 0° gantry and collimator rotation. After machine output validation, calibration strips were irradiated for 6 MV and 15 MV beam energies. To validate the use of a shorter irradiation to scan time (i.e., film development time) than traditionally used for film dosimetry (24 h in our clinic) the 6 MV calibration strips were scanned at 0.5, 1, 2, 3, 4, 5, 6, 12, and 24 h. Our previous study showed EBT4 film's dose response difference between 6 MV and 15 MV beams is less than 2%.^19^ Nevertheless, in the present study, the 15 MV calibration strips were scanned at 24 h and compared to the 6 MV 24‐h scan to further validate the use of the 6 MV calibration curve for both beam energies. The film calibrations (dose vs. optical density) and subsequent dose readings on TBI films were performed using the FilmQA Pro (version 2016) software (Ashland, Bridgewater, NJ).

After calibration, the same setup used for OSLD validation (solid water phantom under TBI conditions) was used to validate film for TBI in vivo dosimetry. First, an ion chamber was placed in the solid water phantom (see Section 2.1) at d_max_, and a fixed 2000 MU was delivered, which approximately equates to a midline dose of 100 cGy in our solid water phantom for an AP/PA delivery. The TBI charge‐to‐dose conversion (see Section 2.1) was used to determine the expected dose at d_max_ to the ion chamber. After ion chamber measurements, the chamber was removed, and two 4 × 4 cm^2^ squares of film were cut, placed under two separate bolus buildup blocks, and placed in the center of the solid water phantom. Diodes were placed around the film blocks to provide a comparison between diodes and film. 2000 MU was delivered, and the film was scanned after 24 h to test the dosimetric accuracy of readout. The dose determined from film was compared to ion chamber and diode readings. This process was repeated for both beam energies, and to validate exit dose measurements for each beam energy.

Finally, end‐to‐end testing was carried out on three patients, where both 4 × 4 cm^2^ film squares and OSLDs (prior to manufacturer recall) were placed under a 1.5 cm‐thick bolus and attached at the six anatomical locations used for TBI in vivo dosimetry at our facility to measure dose during each patient's first treatment fraction. The films were scanned at 4, 5, and 6 h post irradiation, and the film readouts were compared with OSLD readout. TBI film dose at a specific time point was determined using its corresponding time‐specific calibration curve. Note that end‐to‐end testing was only possible for three patients as our OSLD supply was depleted just prior to the recall, and due to the recall, no additional OSLDs were delivered. In addition to the three patients measured with both OSLDs and film, two additional patients were measured with diodes and film to further validate the use of film for in vivo dosimetry.

## 3 RESULTS

### 3.1 OSLD validation

In total, 17 OSLDs were irradiated to create a calibration curve, ranging from 0 to 300 cGy. Three OSLDs were used at each of the three lowest dose levels (0, 5, and 10 cGy), and two OSLDs were used for each of the four higher dose levels (50, 100, 200, 300 cGy). Two validation OSLDs were irradiated, one to 20 cGy and one to 150 cGy, to validate the accuracy of the calibration curve. The measured dose to the low dose validation OSLD was 19.6 cGy, yielding a difference of ‐2.1%, while the measured dose to the high dose validation OSLD was 153.7 cGy, yielding a difference of 2.2%.

After creation of the calibration curve, 12 OSLDs were used to validate the calibration for TBI in vivo dosimetry. Two OSLDs were irradiated to each dose level (150 cGy and 50 cGy for entrance doses, and 100.3 cGy, 80.1 cGy, and 26.7 cGy for exit doses), and the readings of OSLDs were averaged for each dose level to average out uncertainties from individual OSLDs. Measurements were taken for 6 MV and 15 MV beams on the entrance and exit sides of the solid water phantom, and measured doses were compared to expected doses based on calibrated TBI dose rates. Individually, 9 of 12 OSLD readings were within 3.5%. The remaining 3 OSLDs were outside 3.5%. When readings were averaged for each dose level, all OSLDs provided readings within 3.5% of the expected dose.

OSLD entrance and exit dose measurements were also compared to diode measurements under the same conditions. OSLDs and diodes were irradiated to entrance doses of 50 and 150 cGy, and exit doses of 27.6 and 81.7 cGy for a 6 MV beam. Average entrance dose readings for diodes were 49.6 cGy (‐0.8%) and 148.3 cGy (‐1.2%), while average entrance dose measurements for OSLDs were 51.6 cGy (3.2%) and 149.2 cGy (‐0.5). OSLD readings were within 4.0% and 0.7% of the diode measurements for 50 cGy and 150 cGy dose deliveries, respectively. For exit dose measurements, average diode readings were 26.6 cGy (‐2.5%) and 79.9 cGy (‐2.2%), while average OSLD readings were 27.6 cGy (1.4%) and 83.9 cGy (2.7%). OSLD readings were within 4.0% and 5.0% of diode measurements for 27.3 cGy and 81.7 cGy deliveries, respectively.

Finally, OSLD measurements were taken to validate the use of the bolus buildup blocks (as shown in Figure 3c) for in vivo dosimetry. For this test, 50 cGy and 150 cGy entrance doses were delivered under TBI conditions for the 6 MV beam, and OSLD measurements in solid water were compared to OSLD measurements using the bolus blocks. The average difference between the solid water and bolus dose measurements was ‐3.5% for 50 cGy and ‐1.5% for 150 cGy.

### 3.2 Film validation

**FIGURE 4:**
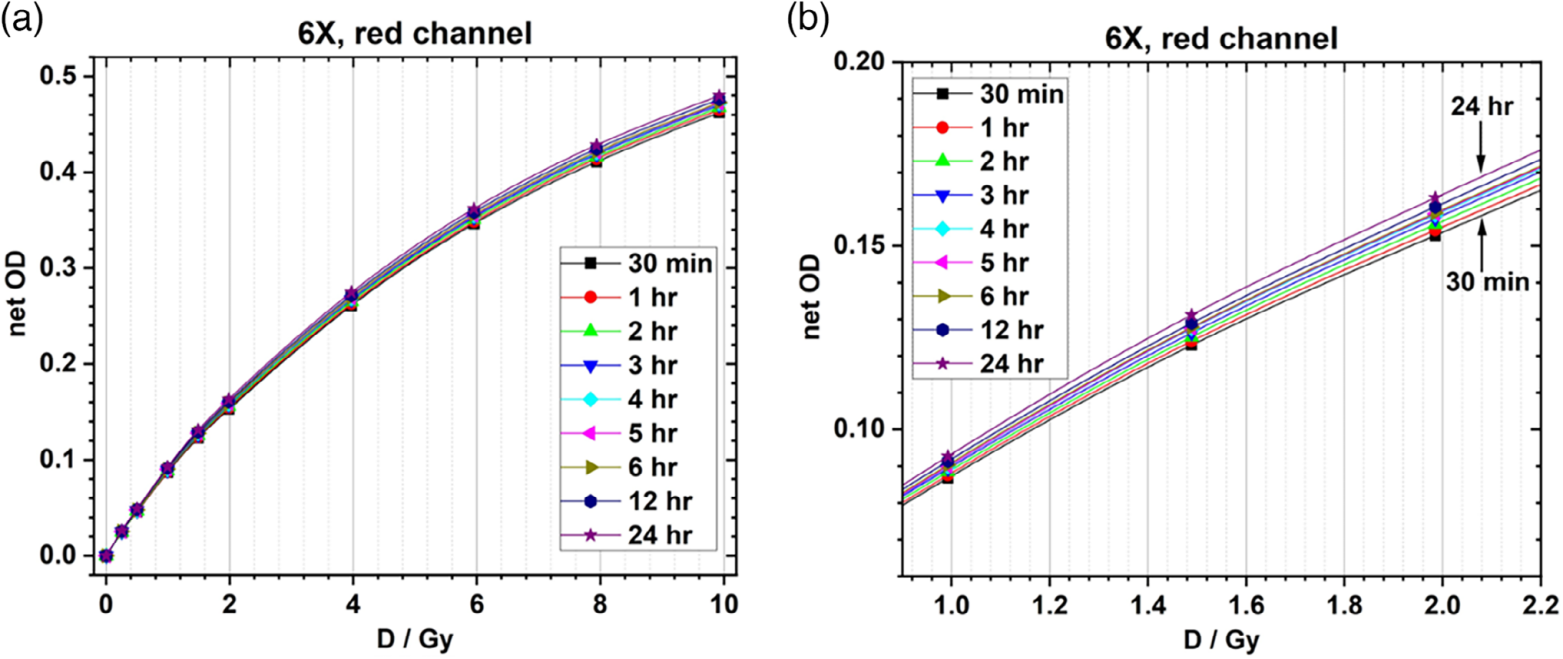
Comparison between calibration curves scanned at shorter development times compared to the curve obtained after 24 h for the 6 MV beam. Full dose range is shown in 4a, while the dose range from ∼1 to 2 Gy is shown in 4b.

**FIGURE 5:**
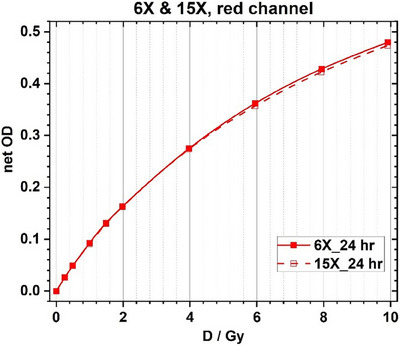
Comparison between 6 MV and 15 MV calibration curves for the red color channel, after 24 h of development time.

Calibration curves were created by irradiating strips of film to different doses, from 0 to 1000 cGy, for 6 and 15 MV beam energies. 6 MV calibration curves were scanned at different time intervals, and the calibration curves from shorter time intervals were compared to the film scan at 24 h to determine if using a shorter development time for in vivo dosimetry was acceptable. Figure 4 shows comparisons between calibration curves scanned at shorter development times compared to the curve obtained after 24 h. Figure 5 shows the comparison between the 6 MV and 15 MV calibration curves, for the red color channel, after 24 h. Note that only the red channel was used for dose determination in this study. In the red‐channel based calibration curves, the dose difference between 6 MV and 15 MV beams is less than 2% at the same net optical density, indicating that the 6 MV calibration curve can be used for film irradiated with the 15 MV beam. This result is consistent with the findings in our previous work.^19^

**FIGURE 6:**
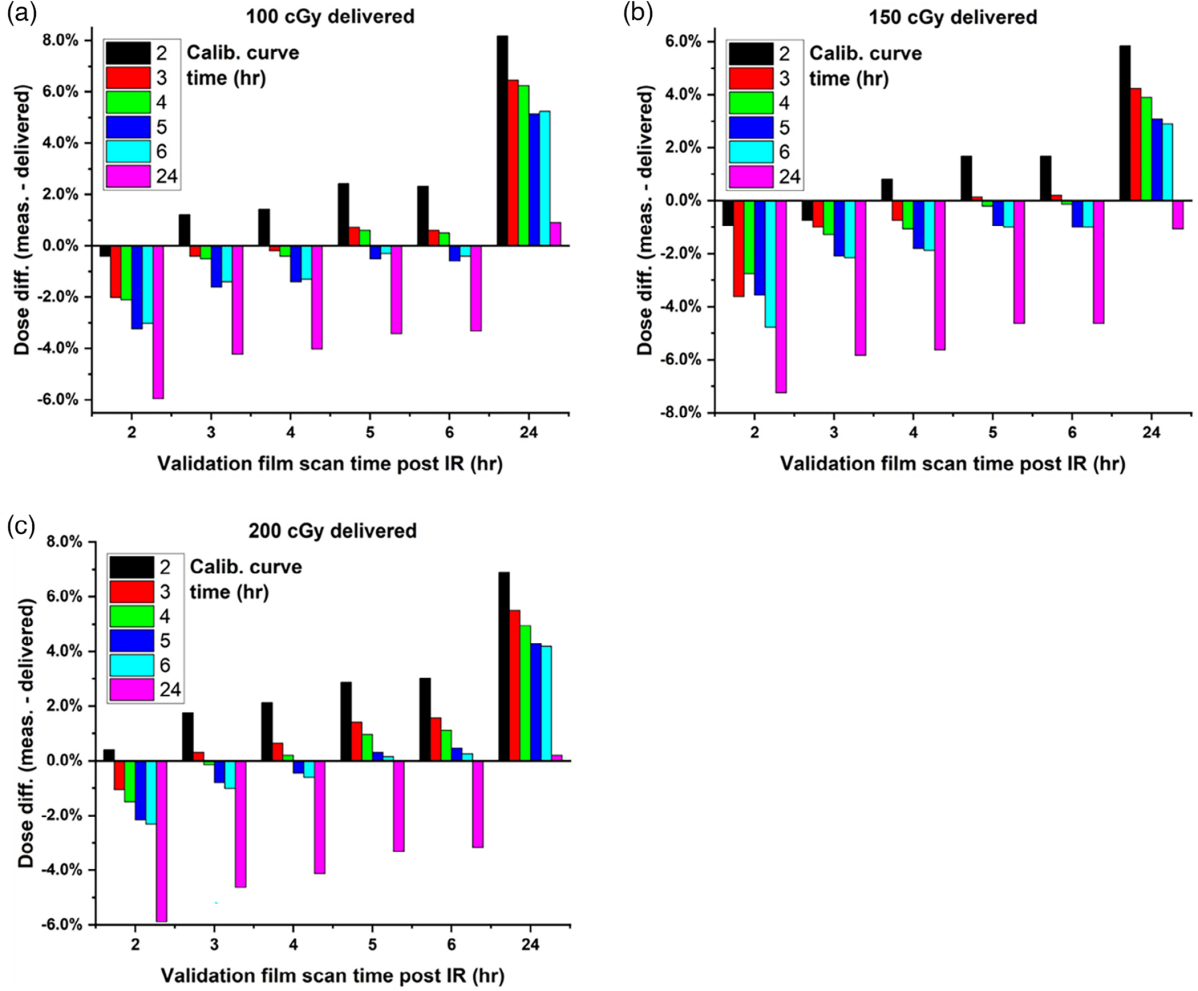
Difference between predicted and measured dose, using calibration curves scanned with increasing development time used to predict doses to films scanned at increasing development times. Differences are shown for delivered doses of 100 cGy (6a), 150 cGy (6b), and 200 cGy (6c). Here the x‐axis represents the time the validation film was scanned, while the color bars represent the calibration curve used to determine film dose.

In order to further tease out any differences between calibration curves, analysis was completed to determine the accuracy of using each calibration curve to determine film doses for different development times. This data is presented in Figure 6. This data indicates that, in general, by using a calibration curve that was scanned after the same amount of time as the experimental films, errors can be maintained within ∼ 1%, and a shorter development time can be used to accurately determine delivered dose to film.

Finally, film measurements under TBI conditions were compared to ion chamber and diode readings for 6 MV and 15 MV beams using a 6‐h development time between irradiation and readout and utilizing the 6‐h calibration curve for the 6 MV beam. Expected entrance doses (determined from the charge‐to‐dose conversion from Section 2.1) were 60.9 cGy and 62.1 cGy for 6 MV and 15 MV, respectively. These compared well with ion chamber measurements of 61.3 cGy and 63.3 cGy for 6 MV and 15 MV, respectively. Entrance dose film readings were within ‐3.2% of ion chamber readings and ‐3.0% of diode readings for the 6 MV beam, and within ‐2.7% of ion chamber readings and 1.1% of diode readings for the 15 MV beam. Expected exit doses were 33.6 cGy and 41.9 cGy for 6 MV and 15 MV, which also compared well to ion chamber measurements of 33.5 cGy and 43.0 cGy for 6 MV and 15 MV. Exit dose film readings were within ‐3.8% of ion chamber and diode readings for the 6 MV beam, and within ‐2.8% of ion chamber readings and ‐3.5% of diode readings for the 15 MV beam.

### 3.3 End‐to‐end patient measurements

End‐to‐end patient measurements are given in Table 1 for three patients evaluated using both film and pre‐recall OSLDs, as well as two patients evaluated using both film and diodes. This table presents the percentage difference between OSLD/diode and film readings for each anatomical location for different film development times, as well as the percentage difference between OSLD/diode readings and the planned dose for each anatomical location. Based on this data, all three calibration curves produce consistent results, with 80% of readings being within 5% of the OSLD/diode measured dose. Note that for the second patient, due to an unusual treatment schedule, film could only be scanned 13.5 h after irradiation (12‐h calibration curve was used). These results compared relatively well with the OSLD readings, with the exception of the neck and ankle film measurements, which deviated by greater than 5% for the 13.5‐h scan. The film scan at 24 h did compare better to the OSLD measurements for these two points. Data from patients 1, 4, and 5 were within 5% of the OSLD or diode readings, while patient 3 had results that deviated >5% from the OSLD readings. OSLD readings for this patient were ∼ 18% low compared to the planned dose, while the film measurements were within 9% of the planned dose.

**TABLE 1:** Percent difference between film and OSLD (patients 1–3) and film and diode (patients 4–5) readings for patient measurements after different film development times for each measurement location. For each patient, the difference between the reference in vivo dosimetry system (OSLD or diode) and the planned dose is shown in the row labeled “Planned."

Patient ID	Scan Time	Forehead	Neck	Umbilicus	Thigh	Knee	Ankle
Patient 1	4 hours	1.4	−0.9	−1.2	−2.0	4.3	2.4
	5 hours	1.2	−0.6	−1.3	−2.4	3.9	2.2
	6 hours	1.7	0.1	−0.8	−2.0	3.8	2.3
	Planned	1.4	7.2	3.8	−4.8	−6.6	−4.7
Patient 2	13.5 hours	−5.0	−5.4	−2.6	−3.8	−1.8	−7.0
	24 hours	−5.1	−5.3	−2.9	−2.1	−1.0	−1.5
	Planned	1.5	−2.8	−1.1	−7.0	−7.5	−3.3
Patient 3	4 hours	5.0	5.6	−2.1	7.1	16.2	12.9
	5 hours	5.5	5.8	−3.8	7.5	16.0	13.1
	6 hours	5.1	5.1	−3.5	6.7	15.2	11.9
	Planned	−8.0	−10.8	2.8	−13.5	−18.4	−18.9
Patient 4	4 hours	−0.4	−3.8	−1.3	−3.7	−0.5	−4.0
	5 hours	−0.3	−3.7	−1.5	−5.0	−0.7	−4.9
	6 hours	−0.1	−3.8	−1.4	−3.9	−0.2	−4.1
	24 hours	−1.3	−3.1	−2.6	−5.0	−1.4	−4.6
	Planned	3.1	3.9	4.7	0.9	−0.5	3.4
Patient 5	4 hours	0.8	−4.6	−1.0	4.0	3.4	1.4
	5 hours	1.4	−4.0	−0.5	4.2	3.7	2.1
	6 hours	0.3	−5.1	−1.2	3.3	3.4	1.5
	24 hours	−0.1	−5.1	−3.0	2.4	3.5	0.2
	Planned	8.0	8.3	7.1	−3.1	−1.0	−0.3

## 4 DISCUSSION

### 4.1 OSLD in vivo dosimetry for TBI

AAPM's TG‐191 report^14^ offers guidelines for the clinical use of luminescent dosimeters, including TLDs and OSLDs. This report offers guidance on calibration, per‐session quality assurance (QA), and annual QA of OSLD systems for high accuracy and high efficiency calibrations. High accuracy calibration should result in uncertainties within 1.6%, while high efficiency calibration should result in uncertainties within 3.5%, for a controlled environment. In our institution, we adhered to the high efficiency workflow, which includes annual readout of standard dosimeters over a range of clinically relevant doses (with absolute dose of standards within 3.4% of the expected dose), annual creation of new constancy dosimeter, annual evaluation of reader sensitivity, and daily QA prior to readout of patient dosimeters.

Our calibration curve was validated using two OSLDs, irradiated to 20 cGy and 150 cGy. The measured doses for these OSLDs were 19.6 cGy (‐2.1%) and 153.4 cGy (2.2%). Given that both validation OSLDs were well within the tolerance given by TG‐191 (± 3.4%), we proceeded with further validation of exit and entrance dose measurements under TBI conditions.

In total, 12 OSLDs were irradiated to validate the calibration curve for TBI in vivo dosimetry. Measurements included both beam energies used clinically, as well as both entrance and exit measurements. OSLD readings were compared to expected doses individually and averaged for each irradiated dose level. Nine of 12 OSLD readings were within ±3.5%. TG‐191 suggests that two‐thirds of the validation OSLDs should be within 3.4%.^14^ Given the overall goal of TBI is to deliver doses within ±10% of the prescription dose, we concluded that having three‐fourths of the validation OSLDs within ±3.5% was acceptable. When OSLD readings from each dose level were averaged, all readings were within ±3.5% of the expected dose.

Utilizing a high efficiency workflow can introduce additional uncertainties into dose measurement. Yoon et al.^20^ have evaluated the sources of error in TBI and total skin electron therapy utilizing OSLDs for in vivo dosimetry in high efficiency settings. Their findings indicate that utilizing vendor provided sensitivities can lead to median uncertainties of ±2.6%, and 95‐percientile error of ±7.1% across OSLD batches. TG‐191 suggests that utilizing vendor provided correction factors for OSLDs can lead to uncertainties in dose readings of ±0.8%.^14^ Changes in SSD can also contribute to uncertainties in OSLD readings, as presented by Ponmalar et al. who found variations of OSLD response of up to 1.5% for SSDs up to 135 cm with 6 MV and 18 MV photon beams.^21^ Given these uncertainties, we utilized two OSLDs per measurement location on each patient and average readings at each location to reduce measurement uncertainty. This workflow was utilized when comparing OSLD measurements to diode and film readings.

Entrance and exit OSLD readings were also validated against diode readings. For the 6 MV beam, entrance and exit dose measurements were within 5% of diode readings for entrance doses of 50 cGy and 150 cGy and exit doses of 27.3 cGy and 81.7 cGy. Estimated dose uncertainty for diodes has been reported to range from 1.5%–3%, while estimated dose uncertainty for OSLDs can range from 2%–3%.^13^ In our measurements, entrance and exit dose readings for diodes were within ± 2.5% of the expected dose, while OSLD readings were within 3.2% of the expected dose. Given the added uncertainties of the high efficiency method, this is in line with the results reported in Minjheer et al.^13^

The results presented here also agree broadly with comparisons completed by other authors. Zhuang et al.^22^ compared OSLD and diode measurements to calculations in the treatment planning system Eclipse (Varian, Palo Alto, CA) for intensity‐modulate radiation therapy plans. They found that diodes and OSLDs were both within 3% of the Eclipse calculated dose, however, they found differences between diode and OSLD readings of up to 14%, though the majority of OSLD readings were within 3.5% of diode readings. Eaton et al.^23^ compared TLD and diode measurements for single fraction TBI patients, and found differences between TLD and diode readings up to ±4.4%, and compared their results to Bloemen‐van Gurp et al.^24^ who compared TLD and MOSFET readings, and found differences up to 12.4%. Finally, Esquivel et al.^12^ compared TLD and OSLD readings, and found differences within ±4.6%.

### 4.2 Film in vivo dosimetry for TBI

Due to manufacturer recall of OSLDs, it became necessary to implement a replacement system for in vivo dosimetry for TBI patients at our center, even though we have the diode system as a backup (to ensure TBI treatment can proceed without interruption in case one of the in vivo dosimetry systems fails). Film was investigated as a second system for in vivo dosimetry. Prior to completing validation measurements with film, it was first necessary to verify that film results could be provided within 4–6 h of irradiation to ensure that physicians had adequate time to review results and time was available if any changes to the compensator or plan were needed before the delivery of the second fraction.

To test the feasibility of reading film dose accurately in a clinically acceptable timeline, we created a calibration curve and scanned the curve at various time points, from 0.5 to 24 h, with calibration curves from different scan times compared to the curve created after 24 h (nominal time between irradiation and scanning used in our department for other patient‐specific QA measurements). Based on Figure 4, visually, it appears that any calibration curve greater than 5–6 h allows us to minimize the uncertainties in the determined dose, while still having a short enough development time to obtain in vivo dosimetry results on a reasonable time scale. However, if we look at each calibration curve's ability to accurately predict dose from films scanned at different time points, we can see that the percentage difference between delivered and measured dose can vary, in some cases more than ± 4% (see Figure 6). Overall, Figure 6 illustrates that errors will be minimized if the development time of the calibration curve matches closely (within approximately 1 h) the development time used for the experimental films. By using a calibration curve that was scanned after the same amount of time as the experimental films, errors can be maintained within ∼ ± 1%, which is well within the accuracy needed for TBI in vivo dosimetry and is similar to the accuracy reported by other authors utilizing OSLDs, diodes, and MOSFETs for in vivo dosimetry.^8^, ^9^, ^10^, ^11^, ^12^

Finally, film measurements under TBI conditions were compared to ion chamber and diode readings for 6 MV and 15 MV beams using a 6‐h development time and utilizing the 6‐h calibration curve for the 6 MV beam. Entrance dose film readings were within ‐3.3% of ion chamber readings and ‐3.0% of diode readings for the 6 MV beam, and within ‐2.7% of ion chamber readings and 1.1% of diode readings for the 15 MV beam. Exit dose film readings were within ‐3.8% of ion chamber and diode readings for the 6 MV beam, and within ‐2.8% of ion chamber readings and ‐3.5% of diode readings for the 15 MV beam.

Su et al.^11^ reported on the use of Gafchromic EBT film (the first model of the EBT film series) for in vivo dosimetry for TBI patients. They completed a comprehensive study utilizing film, ion chamber, and TLDs, and compared readings from film to ion chamber and TLD readings in phantom studies and in patient measurements. They found that the EBT film measurements varied by ‐3.2%–1.5% compared to measurements with an ion chamber in a phantom, while TLDs deviated by ‐4.2%–5.7%. Film readings for phantom measurements were within ‐4.1% and 0.6% of TLD readings. Patient measurements illustrated that film was able to determine patient dose within ± 5% of the prescription dose and reported doses within 5.8% of TLD readings.

Note that the measurements reported in this study were carried out with a single 2000 MU delivery for entrance and exit doses. Using the cGy/MU calibration under TBI conditions, this resulted in expected entrance doses of 61.3 and 63.3 cGy for 6 MV and 15 MV, respectively, and exit doses of 33.5 and 43.0 cGy for 6 MV and 15 MV, respectively. Although the doses delivered to film in this test are lower compared to the dose delivered for TBI patients in our department (100 cGy vs. 150–200 cGy), additional validation tests at higher dose levels were not pursued given that higher dose delivery did yield more accurate results in our calibration curve testing, and the fact that these lower doses were still within ± 4% of the expected delivered dose (comparable to the results of Su et al.^11^). At the TBI treatment dose level, we compared film to OSLD/diode measurement for actual patient cases to get a direct comparison of the systems.

We completed end‐to‐end testing by comparing film and OSLD or diode readings for five patient cases. Patients 1, 3, 4, and 5 had their films scanned at 4, 5, and 6 h, while patient 2, due to an unusual treatment schedule, had their films scanned at 13.5 h. Additionally, films for patients 2, 4, and 5 were also scanned at 24 h. These results are illustrated in Table 1. All three calibration curves produce results within ±5% for the majority of measurements points, in line with the results reported by Su et al.^11^ However, we do see some substantial variation with patient 3, and the neck and right ankle dose for patient 2. The uncertainties in patient 2 may be due to the unusual schedule, however the results from the 24‐h scan indicate that this is likely not the case. For the neck measurement, suboptimal bolus placement may have caused differences between the film and OSLD readings, and inconsistent scanner warmup or region of interest selection during analysis may have contributed to the differences for the ankle measurements. OSLD results for patient 3 were exceedingly low for the right knee and right ankle, with OSLDs reporting doses ∼ 18% lower than the planned dose, while film reported doses ∼ 9% lower. Given that both systems reported low doses for these two anatomical regions, it was determined that the plan needed adjustment to increase the dose to this area. Other than these outlier readings, our measurements agree with those reported by Su et al.^11^

### 4.3 Limitations

This study is not exempt from limitations. One limitation is the use of single channel dosimetry for film analysis. Utilizing dual‐ or triple‐channel methods can reduce uncertainties due to artifacts, variations in the film, or noise within the readout system. In addition, different color channels also offer an extension of the dynamic range to higher doses.^17^ In this study, we utilized the red color channel only. The red color channel calibration curve offered the steepest dose gradient in the dose region of interest (0–300 cGy), giving us the highest sensitivity compared to the other color channels.

A second limitation in this study is the use of a calibration curve extending to 1000 cGy when TBI treatment doses are in the range of 150–200 cGy per fraction. Film dosimetry in our clinic is used for in vivo dosimetry for TBI, as well as patient specific quality assurance. To reduce potential errors in film dosimetry workflows, and to simplify the process for staff, a single calibration curve is used in both instances, and calibration doses cover the range of doses expected for all treatment cases. Data from this study was evaluated with a calibration curve extending from 0 to 400 cGy (low dose calibration curve) to determine the potential uncertainty in using the 0–1000 cGy calibration curve (high dose calibration curve). Variations in doses determined from the high dose calibration curve were within 1.5% of those determined from the low dose calibration curve, apart from one measurement which was within 3% of the dose determined from the low dose calibration curve. Given these small variations, we determined the use of the high dose calibration curve provided sufficient accuracy for TBI in vivo dosimetry.

A third limitation is that a calibration protocol, such as the “one‐scan” method,^25^ was not used in this study. The use of such a calibration protocol can help compensate for the uncertainties in the decreased development time. Due to the limited availability of the machine prior to TBI treatment, this type of protocol was not implemented. Data presented in Figure 6 illustrates that accurate dosimetry is possible (within ∼ ± 1%) by utilizing a calibration curve that matches the development time of the experimental film, therefore this is the workflow we implemented.

Another limitation for this study is the use of small bolus blocks for patient in vivo dosimetry. These bolus blocks do not offer full scatter conditions (as can be seen by the film measurements in Section 3.2) compared to the solid water setup used for calibration. As shown in Section 3.2, film readings collected using these bolus blocks are up to 3.8% lower than ion chamber measurements. However, given the uncertainties in incomplete scatter, the high dose range of the calibration curve, and the use of a calibration curve that matches the development time of experimental films, uncertainties within ∼ ± 4% can still be maintained.

One final limitation relates to how changes in energy spectrum from extended SSD treatment and incomplete phantom scatter within the solid water phantom are handled. The photon beams used in our TBI technique were calibrated under standard TG‐51 reference conditions, with energy‐specific charge‐to‐dose conversion factors used to determine the dose under TBI conditions. The charge‐to‐dose conversion factors could be influenced by changes in photon energy spectrum at the TBI dose measurement points due to photon scattering and the use of beam attenuators (e.g., spoiler or copper compensator). Because the energy spectrum changes from an extended SSD TBI setup are small relative to the energy scale that exhibits appreciable energy dependence for Farmer‐type ion chambers, the chamber calibration factor (thus the charge‐to‐dose conversion) was not adjusted to account for the beam energy spectrum differences.

Likewise, incomplete scatter conditions, like those present with measurements in a small solid water phantom, could impact the in vivo detector calibration. Since the reference dose used to calibrate the in vivo dosimetry systems used in our clinic (diode, OSLD, film) is determined from an ion chamber in the same calibration phantom, phantom size effects are inherently included in the dose measurements for all involved detectors. However, if an in vivo detector's energy response differs from that of the reference ion chamber, increasing phantom size could lead to a change in the calibrated dose response value due to the increase in lower‐energy scattered photons. Since OSLDs and film exhibit minimal energy dependence like ion chambers, increasing phantom size is likely to have a minimal impact on their dose response calibration. Diodes are known to exhibit larger energy dependence; thus, the diode calibration may be impacted further by the phantom size. Further measurements would be needed to determine the impact phantom size has on diode calibration.

## 5 CONCLUSIONS

The results presented in this work illustrate that the latest Gafchromic EBT4 film model can be used for accurate in vivo dosimetry for TBI. Our testing results are in line with those reported previously by Su et al. using the first EBT film model.^11^ Our data indicates that 4–6‐h film development time can give a dose measurement accuracy within ± 4% within a clinically acceptable timeline. We have shown agreement between film, ion chamber, and diode measurements, for 6 MV and 15 MV beam energies, under TBI conditions, of better than 4%. In addition, we have also compared OSLD/diode and film measurements for TBI patients treated within our department. Most of the data points show that film and OSLD/diode readings agree within ±5%, however further investigation into outlier readings is warranted.

## AUTHOR CONTRIBUTIONS

E Draeger contributed to project design, collected and analyzed ion chamber, OSLD, diode, and film data, drafted the manuscript, and revised the manuscript for publication. F Guan collected and analyzed film data, drafted the manuscript, created figures, and revised the manuscript for publication. MY Lee collected and analyzed film data and revised the manuscript. DY Han collected and analyzed ion chamber, OSLD, diode, and film data, and revised the manuscript. W Donahue collected and analyzed ion chamber, OSLD, and diode data, and revised the manuscript. Z Chen provided project design and oversight, data interpretation, and manuscript revisions.

## CONFLICT OF INTEREST STATEMENT

The authors declare no conflicts of interest.
